# First synthesis of a unique icosahedral phase from the Khatyrka meteorite by shock-recovery experiment

**DOI:** 10.1107/S2052252520002729

**Published:** 2020-03-26

**Authors:** Jinping Hu, Paul D. Asimow, Chi Ma, Luca Bindi

**Affiliations:** a Division of Geological and Planetary Sciences, California Institute of Technology, Pasadena, CA 91125, USA; b Dipartimento di Scienze della Terra, Università degli Studi di Firenze, Firenze I-50121, Italy; c Istituto di Geoscienze e Georisorse, Consiglio Nazionale delle Ricerche, Firenze I-50121, Italy

**Keywords:** shock-wave experiments, graded density impactors, icosahedral quasicrystals, Khatyrka meteorite, phase transitions, planetary impacts, nanostructures

## Abstract

Icosahedral quasicrystals (i-phases) in the Al–Cu–Fe system are of great interest because of their perfect quasicrystalline structure and natural occurrences in the Khatyrka meteorite. The natural quasicrystal of composition Al_62_Cu_31_Fe_7_, referred to as i-phase II, is unique because it deviates significantly from the stability field of i-phase and has not been synthesized in a laboratory setting to date. Synthetic i-phases formed in shock-recovery experiments present a novel strategy for exploring the stability of new quasicrystal compositions and prove the impact origin of natural quasicrystals. In this study, an Al–Cu–W graded density impactor (GDI, originally manufactured as a ramp-generating impactor but here used as a target) disk was shocked to sample a full range of Al/Cu starting ratios in an Fe-bearing 304 stainless-steel target chamber. In a strongly deformed region of the recovered sample, reactions between the GDI and the steel produced an assemblage of co-existing Al_61.5_Cu_30.3_Fe_6.8_Cr_1.4_ i-phase II + stolperite (β, AlCu) + khatyrkite (θ, Al_2_Cu), an exact match to the natural i-phase II assemblage in the meteorite. In a second experiment, the continuous interface between the GDI and steel formed another more Fe-rich quinary i-phase (Al_68.6_Fe_14.5_Cu_11.2_Cr_4_Ni_1.8_), together with stolperite and hollisterite (λ, Al_13_Fe_4_), which is the expected assemblage at phase equilibrium. This study is the first laboratory reproduction of i-phase II with its natural assemblage. It suggests that the field of thermodynamically stable icosahedrite (Al_63_Cu_24_Fe_13_) could separate into two disconnected fields under shock pressure above 20 GPa, leading to the co-existence of Fe-rich and Fe-poor i-phases like the case in Khatyrka. In light of this, shock-recovery experiments do indeed offer an efficient method of constraining the impact conditions recorded by quasicrystal-bearing meteorite, and exploring formation conditions and mechanisms leading to quasicrystals.

## Abstract 2

The icosahedral quasicrystal with unique composition Al_62_Cu_31_Fe_7_, from the Khatyrka meteorite, is the only quasicrystalline phase ever discovered in nature prior to being synthesized in a laboratory. This study reproduces this icosahedral phase for the first time using a shock-recovery experiment, proving the planetary impact origin of natural quasicrystals.

## 1. Introduction   

Quasicrystals (QCs) are a unique type of solid characterized by quasiperiodic translational order (Lifshitz, 2003 ▸). The first known QC, for example, has an Al–Mn binary composition and icosahedral symmetry featuring fivefold, threefold and twofold rotation axes (Shechtman *et al.*, 1984 ▸). Since the discovery of the first QC, a number of QCs in Al–TM (transition metal) binary, ternary and quaternary systems have been synthesized at ambient pressure (*e.g.* Tsai, 1999 ▸; Steurer & Deloudi, 2009, ▸ and references therein). In the last decade, the discovery of naturally occurring quasicrystalline phases opened up new questions about QC formation mechanisms under conditions very different from those of conventional metallurgical processing and about the implications of such processes in a geological context. To date, three natural quasicrystalline phases have been identified, exclusively from a single meteorite, the Khatyrka CV3 chondrite (MacPherson *et al.*, 2013 ▸). The first phase is an icosahedral QC (i-phase) with composition Al_63_Cu_24_Fe_13_, officially named icosahedrite (also referred to as i-phase I; Bindi *et al.*, 2009 ▸, 2011 ▸). The second is decagonite (d-phase, Al_71_Ni_24_Fe_5_), named after its decagonal symmetry (Bindi *et al.*, 2015*a* ▸,*b* ▸). Both icosahedrite and decagonite are known to be thermodynamically stable at subsolidus temperatures and were produced by Al-alloy quenching experiments before their discovery in nature (Tsai *et al.*, 1987 ▸; Lemmerz *et al.*, 1994 ▸). Nevertheless, their natural discovery presents a puzzle because the conditions and procedures used in laboratory synthesis of QC from metallic liquid, gas or glass (Tsai, 1999 ▸) hardly resemble any natural rock-forming processes. The third phase is another icosahedral QC but with composition Al_62_Cu_31_Fe_7_ (Bindi *et al.*, 2016 ▸), known as i-phase II. This composition is outside the stability field of icosahedral QCs in the Al–Cu–Fe system and has not previously been produced in any experimental study. It appears that a special formation mechanism or synthesis conditions different from that of classic rapid quenching are needed to understand the occurrence of i-phase II.

The discovery of shock-induced high-pressure silicate minerals in Khatyrka, *e.g.* ahrensite and stishovite, motivated the idea of a planetary impact origin for natural QCs (Hollister *et al.*, 2014 ▸). Subsequently, this idea has been unambiguously supported by successful syntheses of Al–Cu–Fe i-phase and Al–Ni–Fe d-phase by impacting Al alloys in the laboratory (Asimow *et al.*, 2016 ▸; Oppenheim *et al.*, 2017*a* ▸,*b* ▸). Interestingly, the i-phases reported so far from shock-wave recovery experiments have compositions of Al_68–73_Fe_11–16_Cu_10–12_Cr_1–4_Ni_1–2_ (Asimow *et al.*, 2016 ▸), close to but different from any previously observed natural or synthetic i-phases. Hence, although these experiments demonstrate that natural decagonite and i-phases like icosahedrite can have an impact origin, the shock experiments open up two new questions. First, Al–Cu–Fe icosahedrite is of great interest because it has the most perfect (*i.e.* least defective) structure among all known QCs. It also has a quite narrow stability field in composition space, even at the optimal temperature range (550–730°C; Bancel, 1999 ▸). Very small deviations from the stability field lead to complex transformations of the i-phase at lower temperatures (Bancel, 1999 ▸). Nevertheless, the shock-synthesized i-phase has a distinct composition beyond the known stability field and still shows a perfect structure, indicated by robust diffraction studies (Asimow *et al.*, 2016 ▸; Oppenheim *et al.*, 2017*a* ▸). The high pressure and differential stress during shock events may plausibly affect either the (meta)stability or formation mechanism of the i-phase and cause this discrepancy. Second, the usage of stainless steel as a sample chamber and an Fe source in the shock experiments brings Cr and Ni into the system, which also probably affect the stability relations (Oppenheim *et al.*, 2017*a* ▸) and lead to a quinary Al–Cu–Fe–Cr–Ni i-phase. These questions motivated continued studies of QC formation and stabilization by experimental shock compression. One goal is to find an optimal match to the phase assemblages and phase compositions observed in nature, in order to refine estimates of the exact shock conditions that produced the natural QC-bearing assemblage, hence the overall impact history of the Khatyrka meteorite and the origin of extraterrestrial Al–Cu alloys.

In this study, we report two new shock-recovery experiments that used Al–Cu–W graded density impactors (GDIs), initially designed for quasi-isentropic impact loading, as starting materials in the targets. The Al–Cu gradient in a GDI allows sampling of a wide range of Al/Cu ratios and, ideally, traversal of the full stability field of icosahedral Al–Cu–Fe QCs in composition space. In one of the new experiments, we successfully produced Al_62_Cu_31_Fe_7_ i-phase II from reactions in the region that started with a high Cu/Al ratio. We also investigated in detail the associated intermetallic phases, khatyrkite (θ, Al_2_Cu), stolperite [β, Al(Cu,Fe)] and hollisterite (λ, Al_13_Fe_4_), which were either not observed or not fully characterized in previous shock experiments. This is the first laboratory synthesis of i-phase II and the most exact reproduction so far of the complete phase assemblage associated with QCs in the Khatyrka meteorite. The results further reinforce the impact origin theory of natural QC formation and constrain the shock conditions for creating i-phase II and its associated intermetallic companion phases. A complicated pressure–temperature–time path is apparently required to explain the co-existence of i-phase I, i-phase II, decagonite and the assemblage of silicate high-pressure phases observed in Khatyrka.

## 2. Experimental methods   

### 2.1. Graded density impactor   

**Figure 1:**
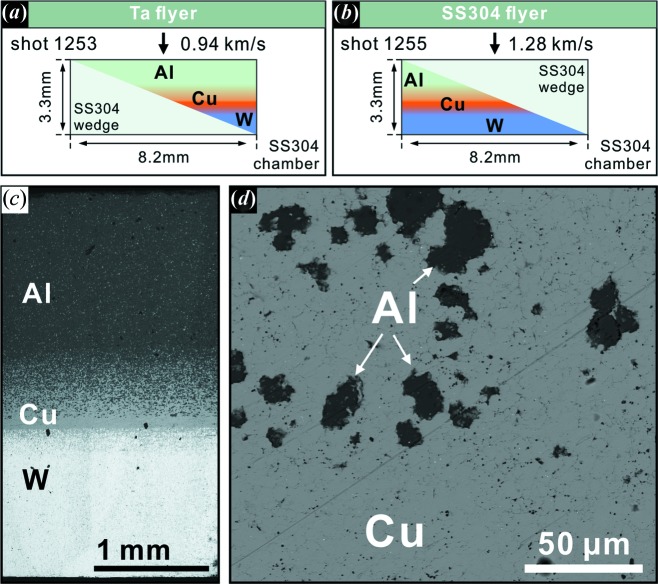
Starting material and target assemblage of the shock-recovery experiments. (*a*), (*b*) The target assemblies of shots 1253 and 1255, respectively. Two wedges cut from one Al–Cu–W GDI disk were used for the two experiments. The size of the SS304 chamber and Ta/steel impactor plate are schematic and are not proportional to the sample size. (*c*), (*d*) SEM BSE image of the cross section of the starting GDI. The Al–Cu gradient is shown in the middle of (*c*). A high-magnification image of the transitional part is shown in (*d*).

The GDI was originally manufactured as a component of gas-gun projectiles to produce quasi-isentropic ramp loading for shock experiments (*e.g.* Kelly *et al.*, 2019 ▸), but was instead used as a target in this study. The Al–Cu–W GDI disk that we used has graded composition from aluminium on top through the full range of Al–Cu alloys to copper in the middle, then through the range of Cu–W alloys to tungsten at the bottom (Fig. 1 ▸). The graded composition is achieved by tape-casting layers of powder mixtures of Al–Cu or Cu–W (Kelly *et al.*, 2019 ▸). The original Al, Cu and W particles are under 325 mesh, *i.e.* 44 µm. The observed particles in the finished fully dense sintered products are mostly equant and ∼20 µm in size [Figs. 1 ▸(*b*) and 1(*c*) ▸]. The overall thickness of the Al–Cu–W GDI disk is 3.3 mm. The Al top is 1.4 mm thick and contains ∼5% of Cu [Fig. 1 ▸(*b*)]. The proportion of Cu particles increases gradually from the Al top towards the 0.1 mm pure Cu layer in the middle [Fig. 1 ▸(*c*)]. Behind the Cu–W transitional zone, the 1 mm W layer on the rear also contains above 5% Cu particles [Fig. 1 ▸(*b*)].

### 2.2. Shock-recovery experiment setup   

The shock-recovery experiments were performed in the Lindhurst Laboratory for Experimental Geophysics at Caltech. We sliced an Al–Cu–W GDI disk at an oblique angle to produce a complementary pair of wedge samples. The original GDI plate was milled to a disk 8.2 mm in diameter and then cut diagonally on a plane inclined 22° from normal to the cylindrical axis into two wedge-shaped halves. The two wedges were used for two separate shock experiments. For each experiment, an identically shaped wedge of 304 stainless steel (SS304) was made to back up the GDI wedge (Fig. 1 ▸) and assemble into an overall right circular cylinder to fit in the sample chamber. The Al top of the GDI faced toward the impact surface of the recovery chamber. The sample assembly was encased in a SS304 chamber and impacted by either a tantalum or SS304 flyer. The chamber and steel wedge operate both as a momentum trap to confine the sample and as an Fe source to the system, which is needed to synthesize Al–Cu–Fe phases.

Although the wedge geometry causes lateral sliding across the wedge interface, the peak pressure experienced by the sample is controlled by (and can be calculated from) the material impedance and is not initially affected by sliding on the wedge interface (Potter & Ahrens, 1994 ▸). The pressures were calculated from the Hugoniots of the metals and alloys using both analytical impedance matching and the *WONDY* 1D hydrocode (Kipp & Lawrence, 1982 ▸). This calculation directly incorporated the porosity, density, sound speed and shock impedance of the GDI as reported in the work by Kelly *et al.* (2019 ▸).

The first experiment, shot 1253 (S1253), used the Al-rich half of the GDI and a tantalum flyer. The impact velocity of 0.93 km s^−1^ produced an estimated peak shock pressure of 20–30 GPa for 800 ns in the sample (see Fig. S1 in the supporting information). In the two-wedge sample geometry, the pressure–time history varied both along the shock direction and perpendicular to it as the layer thicknesses changed. The Al-rich thin end of the GDI wedge [left side of Fig. 1 ▸(*a*)] has a lower shock impedance than the steel chamber and was not backed by the Cu–W layers. It was initially compressed to 14 GPa by the first shock front and then reshocked to 23 GPa by a wave reflected from the steel chamber. In contrast, the thicker part of the wedge [middle right of the GDI in Fig. 1 ▸(*a*)] had layers of Cu–Al (and Cu–W). This part was first shocked to ∼21 GPa and gradually compressed to >25 GPa as the shock wave traversed the Cu–W gradient (Fig. S1). Only the thickest part of the wedge [right side of Fig. 1 ▸(*a*)], with full Cu–W layers, experienced the 30 GPa peak pressure. Because the Cu–W regions have higher impedances than the steel chamber, there was no reshock; rather a partial rarefaction wave propagated back into the sample after the first shock front reached the rear sample-chamber interface. This rarefaction wave subsequently intersected release waves from the sidewall and from the back of the flyer to create a spatially and temporally complex pressure-release pattern. In the thickest part of the GDI, it took 1 µs for the peak pressure to drop to 10 GPa and 1.7 µs to release to 0 GPa (Fig. S1). The thinner part of the wedge saw a peak-pressure pulse shorter than 800 ns and a faster release. The second experiment, S1255, used the W-rich half GDI for a sample and a SS304 flyer with an impact velocity of 1.28 km s^−1^ (Fig. 1 ▸). The corresponding peak pressure was 30–35 GPa for 600 ns. Since this wedge had a uniform W-rich back, the peak pressure was also relatively uniform.

The finely graduated Al–Cu transition in the GDI provides an efficient way to sample a full range of Al/Cu ratios in the starting material in one experiment. The wedge sample is designed to convert the different particle velocities across the GDI/steel interface into a component of interface-parallel sliding and thus create strong shear flow. The sheared zone is expected to enable or enhance melting and simultaneous reactions at the GDI/steel interface. We emphasize that, contrary to many shock-recovery capsules, this experiment is not designed either to maintain simple 1D particular motion across a planar shock front or to allow enough time for full reverberation across the sample to bring the pressure to a value equilibrated with that in the chamber walls.

### 2.3. Sample preparation and analytical techniques   

**Figure 2:**
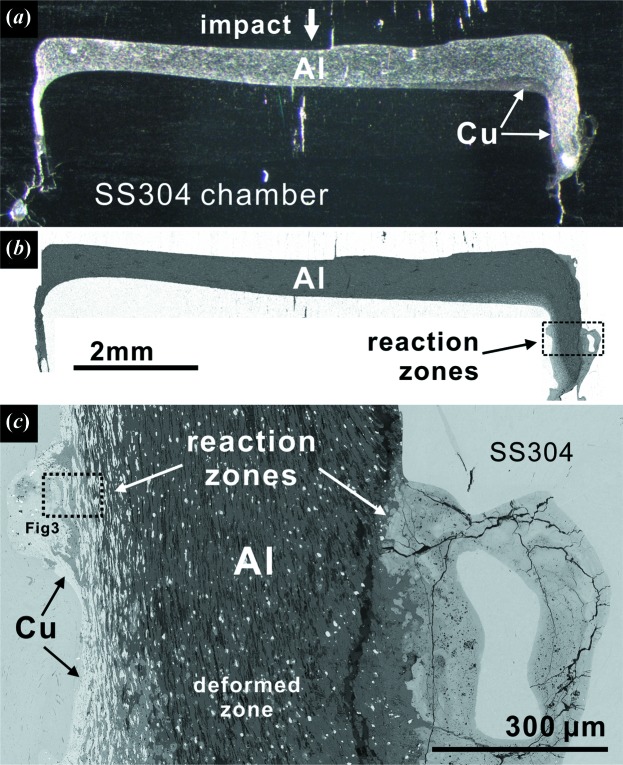
The overall image of the shocked GDI wedge of S1253 and its deformed zones. (*a*), (*b*) represent reflected light and BSE images of the sample. The ramp of the wedge is deformed into an L shape and reaction is concentrated along the right wall. (*c*) A BSE image of the boxed area in (*b*). Reactions between the GDI and the steel chamber occur along the right and left edges of the GDI material, which began before deformation as the Al top and Al–Cu ramp of the GDI, respectively. The boxed area is shown in Fig. 3 ▸.

The recovered sample was cut through the mirror plane of the GDI wedge [Fig. 2 ▸(*a*)]. The exposed surface was polished on diamond lapping films and analyzed with a field-emission scanning electron microscope (FE-SEM) in the Division of Geological and Planetary Sciences at Caltech. Backscattered and secondary electron (BSE and SE) images were employed to observe the microtextures of the run product. Energy-dispersive X-ray spectroscopy (EDS) with a silicon drift detector was used to measure the chemical composition of the intermetallic phases. To accurately measure the compositions of submicrometre grains of interest, we collected and compared spectra obtained by operating the SEM at 10, 12, 15 and 20 kV accelerating voltage and 4–6 nA beam current. The corresponding X-ray excitation volumes in Al–Cu–Fe alloy range from ∼0.5 to 1.8 µm in both depth and diameter, indicated by Monte Carlo simulations (Gauvin *et al.*, 2006 ▸). Each EDS spectrum was collected for 10 s with more than 200 counts channel^−1^ and 40% dead time. Our previous study indicates that EDS measurement of intermetallic phases in this size range with this protocol is accurate and in good agreement with electron-microprobe quantitative analysis (Oppenheim *et al.*, 2017*a* ▸). We employed electron backscatter diffraction (EBSD) to determine the (quasi)crystal structure of the phases. Kikuchi bands in the diffraction patterns of the crystalline phases are indexed to lattice planes, with mean angular-deviation values of less than 0.6°. The icosahedral QC phases, which cannot be indexed by the *AZtec* EBSD nanoanalysis software (Oxford instruments) despite high pattern quality, are identified from the arrangement of fivefold, threefold and twofold rotation axes in the diffraction patterns.

## 3. Results of shock-wave experiment   

### 3.1. Shock deformation in experiment S1253   

Overall, shock-induced deformation is concentrated along the ramp and at the corners of the GDI wedge and identified from the significantly changed shape (Fig. 2 ▸) compared with the starting wedge (Fig. 1 ▸). Shock-shear melting and reactions between Al, Cu and steel are associated with the deformation zones and produce new intermetallic phases [Fig. 2 ▸(*c*)]. The long interface between the Al-rich top of the GDI and steel driver is coherent and well defined without much reaction. In contrast, the bottom portion of the wedge is deformed into an L shape [Figs. 2 ▸(*a*) and 2 ▸(*b*)], significantly different from the starting triangular cross section of the wedge [Fig. 1 ▸(*a*)]. In the lower-right corner (Fig. 2 ▸), the sample has been squeezed and extruded along the direction of the impact, making it normal to the impact face and the coherent Al-rich top of the wedge. In this region of high strain, both the GDI and the steel chamber (or insert) underwent partial melting, with simultaneous reaction between them [Fig. 2 ▸(*c*)]. In Fig. 2 ▸(*c*), the compositional gradients have been rotated to the horizontal direction by the deformation. The right side is Al rich and forms relatively Cu-deficient intermetallic phases by reaction with steel. The left side has a higher Cu/Al ratio and forms Al–Cu–Fe phases that resemble the natural metallic assemblage in the Khatyrka meteorite, including i-phase II. Details of these reacted assemblages are described in the following sections.

### 3.2. i-phase II assemblage in S1253   

**Figure 3:**
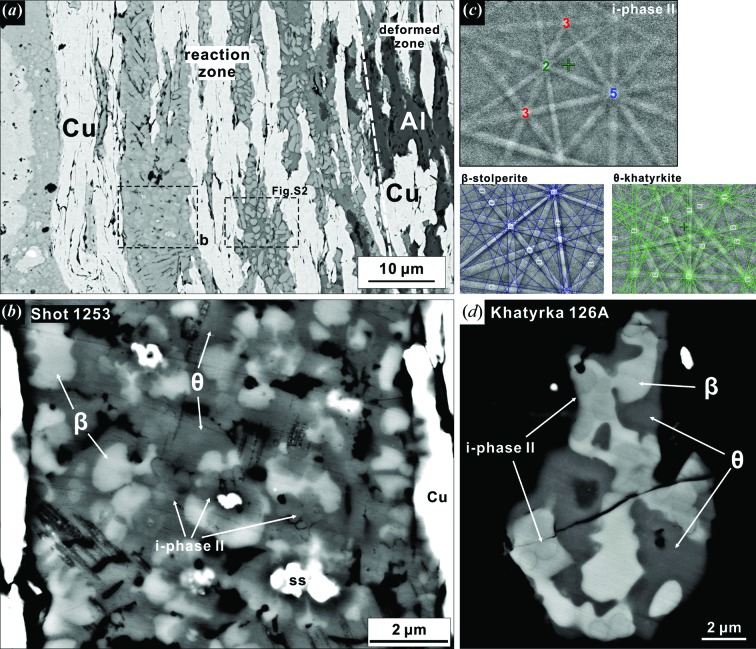
BSE images of the QC-bearing assemblage from the reaction zone in S1253. (*a*) The region from the boxed area in Fig. 2 ▸(*c*). The right side of the image presents deformed and elongated Al–Cu grains from the GDI. The left side shows the texture of reaction products between the GDI and the steel chamber. The QC assemblage is shown in (*b*) and the intermetallic assemblage is shown in Fig. S2. (*b*) The assemblage of i-phase II, khatyrkite (θ), and stolperite (β). The i-phase II forms petal-like domains. Stolperite is brighter and more euhedral than khatyrkite. (*c*) EBSD patterns of the identified phases. The coloured numbers mark the rotation axes of icosahedral symmetry in the i-phase II pattern. Coloured lines denote the indexed poles of the Kikuchi bands. (*d*) The same i-phase II assemblage in a natural sample, Khatyrka meteorite grain 126A [after the work by Bindi *et al.* (2016 ▸)]. The i-phase II in this case is enclosed by stolperite.

Reactions between the steel chamber and the Al–Cu mixture on the Cu-rich side produced intermetallic phases in association with deformed Cu grains [Fig. 3 ▸(*a*)]. Away from the zone of melting and extensive reaction, the remnant Al and Cu have been deformed into 10 × 40 µm elongated grains [Figs. 3 ▸(*a*) and S2(*a*)] from the starting ∼20 µm equant grains [Figs. 1 ▸(*b*) and 1 ▸(*c*)]. Reaction in this region is limited to narrow (<1 µm) bands along the Al–Cu grain boundaries. In contrast, in the reaction zone, the aluminium is probably consumed by eutectic melting (Lin *et al.*, 2017 ▸; Suttle *et al.*, 2019 ▸) and completely reacted with Cu into the intermetallic phases [Fig. S2(*a*)], whereas Cu is partially remnant. The stainless-steel chamber is locally incorporated in the reaction as the only Fe source.

Al–Cu–Fe icosahedral QCs occur only in specific locations with Cu/Al > 1 and are associated with reactions involving the steel chamber. Fig. 3 ▸(*b*) demonstrates the texture of the icosahedral QC and associated phases. The grains with medium grey contrast and semi-radial patterns represent i-phase II (see below). The EBSD pattern of these grains [Fig. 3 ▸(*c*)] includes fivefold, threefold and twofold rotation axes demonstrating icosahedral symmetry. High-contrast Kikuchi bands in the pattern indicate the robustness of the QC structure. The diffraction also indicates that the core and petals of a given i-phase aggregate have the same crystallographic orientation. That is, each contiguous i-phase grain is a single QC domain. Because of the small domain size, we used various accelerating voltages (10, 12, 15 and 20 kV) to image and analyze nine well defined QC domains (Figs. S2 and S3 and Table S1 in the supporting information). The formula of the i-QC averaged from 10 kV analyses is Al_61.5_Cu_30.3_Fe_6.8_Cr_1.4_ with an uncertainty of up to 2.8 atomic percent (Tables 1 ▸ and S1). The results from 12 and 15 kV analyses are identical to 10 kV analyses within the uncertainty (Table S1), whereas a few 20 kV analyses show clear interference from surrounding phases. This composition is in good agreement with natural i-phase II Al_62_Cu_31_Fe_7_ and is distinct from the Al_63_Cu_25_Fe_12_ i-phase I in the Khatyrka meteorite (Tables 1 ▸ and S4). The shock-synthesized i-phase II also contains minor Cr because the Fe source in this experiment is the stainless-steel chamber.

Two intermetallic phases are associated with i-phase II in the reaction zone [Fig. 3 ▸(*b*)]. The phase with dark grey BSE contrast, close to that of i-phase II, shows a tetragonal symmetry matching the *I*4/*mcm* space group in its EBSD pattern [Fig. 3 ▸(*c*)], identified as khatyrkite (also referred to as the θ phase in the Al–Cu–Fe system). The 1 µm grain size of khatyrkite is sufficient for robust EDS analysis (Table 1 ▸). Its formula of Al_65.1_Cu_34.6_Fe_0.3_ also matches with natural khatyrkite, with slight Al deficiency relative to the ideal formula Al_2_Cu of the θ phase. Khatyrkite is not a significant Fe host but it contains a few atomic percent more Cu than i-phase II (Table 1 ▸), leading to a similar mean atomic number and an almost identical BSE contrast. The light grey phase in the BSE images is stolperite [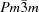, known as the β phase, ideal formula Al(Cu,Fe)], identified by EBSD [Fig. 3 ▸(*c*)] and EDS analysis (Table 1 ▸). Stolperite generally provides very good band quality in EBSD patterns that allows unambiguous differentiation of the primitive lattice from the body-centred cubic lattice (Oppenheim *et al.*, 2017*a* ▸). Its formula of Al_57.3_Cu_40.6_Fe_1.6_Cr_0.4_Ni_0.1_ is again nearly identical to the natural occurrence in the Khatyrka meteorite, except for minor Cr and Ni from the steel (Table 1 ▸). Besides the i-phase II + khatyrkite + stolperite assemblage, there are fragments in the reaction zone with bright white BSE contrast [Figs. 3 ▸(*b*) and S3]. These are remnant stainless-steel fragments, proving the inflow of the chamber material to the reaction zone.

In this i-phase II + khatyrkite + stolperite assemblage, none of the mineral grains are completely euhedral except that the petal texture of the i-phase II can be considered as a hint of its symmetry [Fig. 3 ▸(*b*)]. The stolperite occurs as subhedral rounded grains that show a subtle tendency to enclose the i-phase II grains, suggested by the concavity of stolperite-QC interfaces. In contrast, the khatyrkite is mostly anhedral and interstitial to the i-phase II and stolperite. This is noticeably different from the khatyrkite predominant assemblage described in the following section.

### 3.3. Non-QC assemblage in S1253   

Apart from the i-phase II assemblage described above, the majority of the reaction zones in S1253 [Figs. 2 ▸(*c*) and S4(*a*)] contains only Al–Cu–Fe intermetallic phases without QCs. Figs. 3 ▸(*a*) and S2(*c*) show a typical reacted area adjacent to the i-phase II assemblage. In this area, submicrometre grains occur in a matrix of finer-grained material of darker BSE contrast. EBSD and EDS analyses indicate that the coarser grains are khatyrkite (Table S2). The formula Al_67–71_Cu_29–33_ matches with the ideal θ phase and no Fe can be detected. Equant, elongated and irregular grains of khatyrkite are all common in this region, with subhedral shapes surrounded by the matrix [Fig. S2(*c*)]. The matrix contains grains smaller than 100 nm, which is difficult to analyze with the SEM. Based on their BSE contrast, the matrix phases are inferred to be pure Al plus another Al-rich alloy. It is worth noting that neither steel fragments nor measurable concentrations of Fe, Cr or Ni occur in these khatyrkite predominant regions, in contrast with the i-phase II region [Figs. 3 ▸(*a*) and S2].

As shown in the overall image of the sample (Fig. 2 ▸), the right side of the GDI was deformed along the impact direction and both top and bottom [correspondingly right and left in Fig. 2 ▸(*c*)] of the GDI melted and reacted with the steel insert or chamber. Although deformation in the top and bottom part is equally strong, the resulting reactions and product phases are quite different. The QCs only occur along the Cu-rich bottom of the ramp. Fig. S4 shows the textures of the complementary Cu-deficient phases along the Al-rich top. In this reaction region [Fig. S4(*b*)] there are portions with distinctively high and low BSE contrast, corresponding to high and low iron content. The low-contrast portion has two phases, a black granular phase and a dark grey dendritic phase, in the BSE image [Fig. S4(*b*)]. The phase with black contrast is pure Al, probably recrystallized to submicrometre domains from the large starting Al grains. The dendrites are hollisterite (*C*2/*m*, referred to as the λ phase) based on EBSD and EDS analyses (Table S2). The formula Al_64.7_Fe_18.7_Cu_8.1_­Cr_5.5_Ni_2.5_Mn_0.5_ includes significant Cu compared with the ideal formula Al_13_Fe_4_ (that is, Al_76.5_Fe_23.5_ on a 100-atom basis) but is in good agreement with the natural hollisterite in Khatyrka (Ma *et al.*, 2017 ▸). Distinctively, the portion with high BSE contrast is dominated by petal-textured grains [Fig. S4(*c*)]. This is another β phase with a more Fe-rich formula (Al_49_Cu_7.6_Fe_30.3_Cr_9_Ni_3.2_Mn_0.5_Si_0.3_, Table S2) than the stolperite in the i-phase II region; we refer to this as Fe-stolperite. The aggregates of the petals are 3–5 µm in size and each petal/dendrite is submicrometre. In this area, Fe-stolperite also occurs as equant grains without forming aggregates [Fig. S4(*c*)]. There are small voids spreading through the Fe-stolperite region, appearing as black dots in the BSE image. Generally, the reaction zone along the Al-rich top of the GDI incorporated a larger fraction of the steel chamber than the i-phase II assemblage along the opposite chamber wall, as shown by positive correlation between Cr, Fe and Ni. Mn and Si can also be detected by EDS in reacted phases in this area (Table S2).

### 3.4. Deformation and icosahedral QC in S1255   

**Figure 4:**
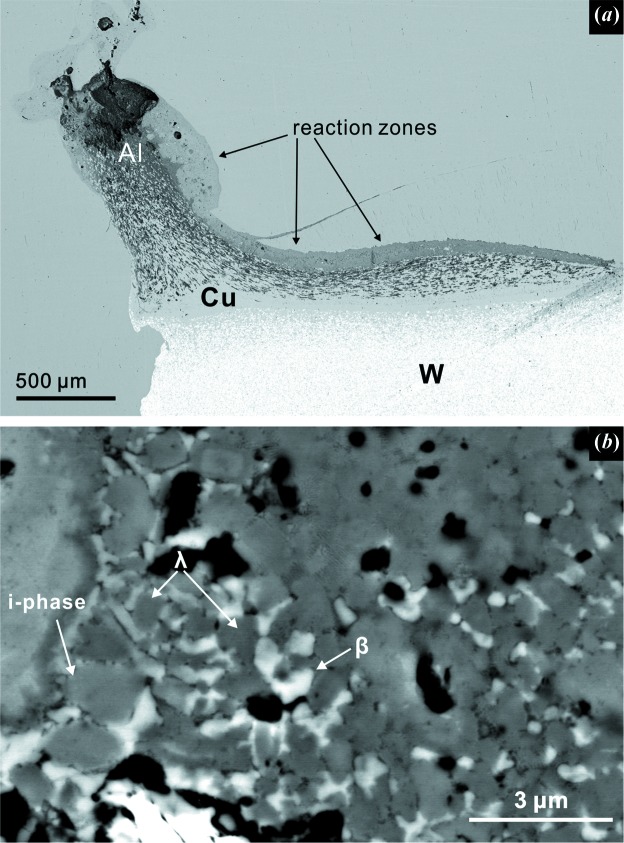
BSE images of S1255. (*a*) The overall image of the shock-deformed GDI. The grey smooth background is the steel chamber. Reactions occur continuously along the Al–Cu ramp. (*b*) A representative zoomed-in image of the phase assemblage in the reaction zone in (*a*). The i-phase and hollisterite (λ) occur as subhedral grains, while stolperite (β) is more anhedral.

The GDI wedge used in S1255 has a ramp with an Al–Cu gradient and a W base, which provides a higher shock pressure than S1253 [30 GPa (Fig. S1)]. It is easy to distinguish the run product of S1255 by the continuous reaction zone along the Al–Cu ramp [Fig. 4 ▸(*a*)]. The concave shape of the deformed ramp matches the impact direction, whereas the top corner preserves the ramp angle. This suggests weaker local deformation than in S1253, where part of the ramp was fully transposed to the vertical direction.

**Figure 5:**
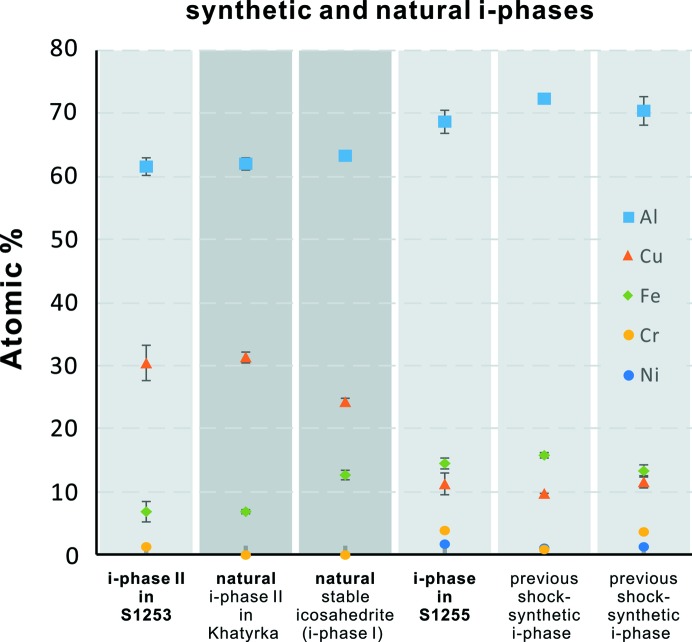
Compositions of the natural and synthetic i-phases. Corresponding EDS analysis results are shown in Table S4. The error bar is the standard deviation of multiple individual analyses. When there is no symbol for a certain element it means the element is not detected in the analysis. When there is no visible error bar it means it is smaller than the size of the symbol. References: natural i-phase II in Khatyrka, Bindi *et al.* (2016 ▸); natural thermodynamically stable icosahedrite (i-phase I), Bindi *et al.* (2011 ▸); a previous shock-synthetic i-phase (second from the right), Asimow *et al.* (2016 ▸); and a previous shock-synthetic i-phase (far right), Oppenheim *et al.* (2017*a* ▸).

The phase assemblage in the continuous reaction zone of S1255 is relatively consistent (except at the Cu-free pure Al tip). Unlike the limited occurrence of i-phase II in S1253, icosahedral QC is a major constituent of the reaction zone of S1255. Fig. 4 ▸(*b*) demonstrates a representative zoomed-in image of the reaction region. The i-phase is identified by EBSD patterns matching icosahedral symmetry. In this case, the i-phase mostly occurs in angular but equant grains up to 3 µm in size. QC aggregates are not observed in the S1255 reaction zone. The i-phase formula, Al_68.6_Cu_11.2_Fe_14.5_Cr_4_Ni_1.8_ (Table S3) is quite distinct from either the i-phase II in S1253 (Al_61.5_Cu_30.3_Fe_6.8_Cr_1.4_) or the optimal i-phase (icosahedrite, Al_63_Cu_24_Fe_13_), but is similar to the previously shock-synthesized i-phase (Fig. 5 ▸ and Table S4). The reaction zone contains two more major phases in direct contact with the i-phase. First, the phase with very slightly darker BSE contrast than the i-phase [Fig. 4 ▸(*b*)] is identified as hollisterite (λ, Al_13_Fe_4_), again by EBSD and EDS (Table S3). The grains are mostly equant, granular and subhedral, but some rectangular grains of hollisterite manifest the prismatic euhedral crystal form. The formula, Al_70.4_Cu_10.6_Fe_12.7_Cr_3.9_Ni_1.4_, generally matches with the hollisterite in Khatyrka (Ma *et al.*, 2017 ▸) and S1253, with some Fe deficiency (Table S3). Second, EBSD and EDS indicate that the light grey phase in the BSE image [Fig. 4 ▸(*b*)] is a β phase. The grains are mostly angular and anhedral. The composition of this β phase in S1255 varies by up to 20 at.% in Cu and Fe, resulting in a formula range of Al_47–58_Cu_14–34_Fe_12–23_Cr_3–7_Ni_1–3_, whose projection into the Al–Cu–Fe ternary plots along a binary join between ideal AlCu (stolperite) and ideal AlFe. Despite the compositional variation, the quality of the EBSD patterns of this phase are consistently robust, matching the primitive cubic structure of the β phase.

## 4. Discussion   

### 4.1. Composition and stability of quaternary/quinary shock-synthesized i-phases   

The i-phase II in S1253 is the first shock-synthesized QC that almost exactly matches the composition of a natural i-phase (Table 1 ▸ and Fig. 5 ▸). This is a composition never seen before in laboratory syntheses and is outside the well defined stability field of the i-phase at ambient pressure. The natural i-phase II in Khatyrka occurs in domains that are >1 µm in size [Fig. 3 ▸(*d*)], allowing for robust *in situ* chemical analysis, even though the occurrences are limited to only a few areas of a single fragment of the meteorite. In the case of Khatyrka, more than ten electron-microprobe analyses provide a consistent composition of Al_61.9_Cu_31.2_Fe_6.8_Cr_0.1_, with less than 1 at.% uncertainty (Table 1 ▸; Bindi *et al.*, 2016 ▸; Lin *et al.*, 2017 ▸). The i-phase II in S1253, however, is more challenging to analyze because of its submicrometre domain size [Figs. 3 ▸(*b*) and S3]. The low accelerating voltage (10 kV) FE-SEM-EDS analysis helps by reducing the excitation volume significantly to ∼0.5 µm in depth and diameter. The average composition from low-voltage analysis is Al_61.5_Cu_30.3_Fe_6.8_Cr_1.4_ with uncertainty (standard deviation) on the four elemental atomic percentages of 1.33, 2.82, 1.69 and 0.34 at.%, respectively (Table 1 ▸). Measurements on the same individual grains with up to 20 kV voltage (up to 1.8 µm in depth and diameter of the excitation volume) result in the same level of uncertainty. This suggests that the deviation is not primarily caused by random error in the low voltage/current measurement but rather records small compositional variation among the analyzed i-phase II grains (Table S1). Nevertheless, the 15–20 kV analyses, with excitation volumes larger than the grains, start to show moderate interference from surrounding khatyrkite and stolperite [Figs. S2(*b*) and S3]. This is consistent with the fact that the 20 kV BSE image shows blurred grain boundaries (Fig. S3). However, in the 10 kV BSE image the grain boundaries are sharp and clear. Because the majority of the 10–12 kV analyses show consistent results, we take the 10 kV average as the best estimate of the composition of synthetic i-phase II in S1253 (Table 1 ▸). This formula, Al_61.5_Cu_30.3_Fe_6.8_Cr_1.4_, is a good match with natural i-phase II Al_61.9_Cu_31.2_Fe_6.8_Cr_0.1_ and distinct from the thermodynamically stable icosahedrite (Al_63_Cu_24_Fe_13_; Bindi *et al.*, 2011 ▸), even when considering the 1–2 at.% uncertainty (Fig. 5 ▸).

Another distinctive feature of the shock-synthesized i-phase is the incorporation of Cr and Ni from the stainless-steel chamber (Fig. 5 ▸). The i-phase II in S1253 contains 1.4 at.% Cr versus 0.1% in natural i-phase II. Generally, the Cr and Ni content of the S1253 and S1255 i-phases analyses increase with Fe content and can contain up to 4% Cr and 1.8% Ni (Fig. 5 ▸ and Table S4). Their corresponding Cr/Fe ratios fall between 0.2 and 0.3, and the Ni/Cr ratios are ∼0.5 (Table S4). These ratios agree with the 18/8 (18% Cr and 8% Ni) composition of SS304 from the sample chamber, which is the only Fe source. This suggests that Cr and Ni are neither selectively taken up by the QC in preference to Fe nor selectively excluded. Nevertheless, the Cr + Ni content may affect the Al/Cu ratio in the Al–Cu binary cluster in the i-phases. Oppenheim *et al.* (2017*a* ▸) studied the stability of the Al–Cu–Fe–Cr–Ni quinary i-phase, using the Hume–Rothery theory based on a valence electrons per atom criterion. This theory suggests that >70% Al and equal Cu–Fe contents are preferred if the i-phase is to absorb >5% Cr + Ni. This helps explain the unexpectedly high Al content (>68 at.%) and nearly equal Fe and Cu contents of all the shock-synthesized i-phases in S1255 and in previous studies (Fig. 5 ▸). In contrast, the Cr and Ni in the measured i-phase II are low or even undetectable in some grains (Fig. 5 ▸), which may be one of the reasons that its Al and Cu/Fe content are such a close match to natural i-phase II. Moreover, Wolf *et al.* (2019 ▸) analyzed semi-equilibrated Al–Cu–Fe–Cr samples made by co-sputtering followed by quick annealing and observed a high Cr i-phase with Al_63–65_Cu_12–22_Fe_5–11_Cr_6–15_ composition. This suggests that metastable i-phases can incorporate significant Cr. The stability of i-phase II in contact with intermetallic assemblages in the Al–Cu–Fe system will be discussed in the next section.

### 4.2. Formation and stability of i-phase and intermetallic co-existing phase assemblage   

There have been a number of studies on the phase boundaries of i-phases in the Al–Cu–Fe ternary system since its first synthesis (Tsai *et al.*, 1987 ▸). Previous work investigated the stability field for both subsolidus (*e.g.* Bancel, 1999 ▸) and liquidus (*e.g.* Zhang & Lück, 2002 ▸, 2003 ▸; Stagno *et al.*, 2017 ▸) conditions. It has been found that only a very narrow range of composition in the vicinity of the optimal Al_63_Cu_24_Fe_13_ is thermodynamically stable through the whole subsolidus temperature range (Bancel, 1999 ▸). In this sense, it is not surprising that the first discovered natural i-phase, induced by impact, sits right on this optimal composition and survived a complex series of planetary and terrestrial processes (Bindi *et al.*, 2009 ▸, 2012 ▸; Lin *et al.*, 2017 ▸). In contrast, this restricted stability range for the i-phase also makes it challenging to understand the conditions of formation of i-phase II and other shock-synthesized i-phases.

Since S1253 almost exactly reproduced the phase assemblage, composition and texture of the i-phase II in the Khatyrka meteorite (Fig. 3 ▸; Table 1 ▸), their conditions and sequence of formation can be discussed together. The i-phase II can occur either as a stable or metastable phase; evidently, the former would require a thermodynamic stability field. A simple but important fact is that a typical shock-recovery experiment produces a high-pressure pulse of ∼1 µs duration (Fig. S1) and a shock temperature of a couple of 100°C in dense metal samples at 20–30 GPa (Oppenheim *et al.*, 2017*a* ▸). This temperature–time condition cannot drive long-range diffusion or reconstructive solid-state transformations in metals and alloys (Porter *et al.*, 2009 ▸). Therefore, reaction and new intermetallic phases need to occur either in areas of local melting by strong shear deformation and collapse of pre-existing pores and voids from shock compression (Oppenheim *et al.*, 2017*a* ▸) or by a much slower process after release from the shock state. The latter is not favoured because, in shots 1253 and 1255, the melting and reaction zones are exactly coincident with the regions of strongest deformation, which are localized at ramp corners and edges. The central coherent part of the GDI is compressed and released uniaxially without generating new phases (Fig. 2 ▸). In this scenario, the liquidus phases are the most likely to occur in the reaction zones and could then quench through heat transfer to the colder surroundings with the possibility of undergoing subsequent peritectic or subsolidus transformations as they cool. Therefore, both liquidus and solidus phase relations are relevant in explaining the stability of the i-phases.

**Figure 6:**
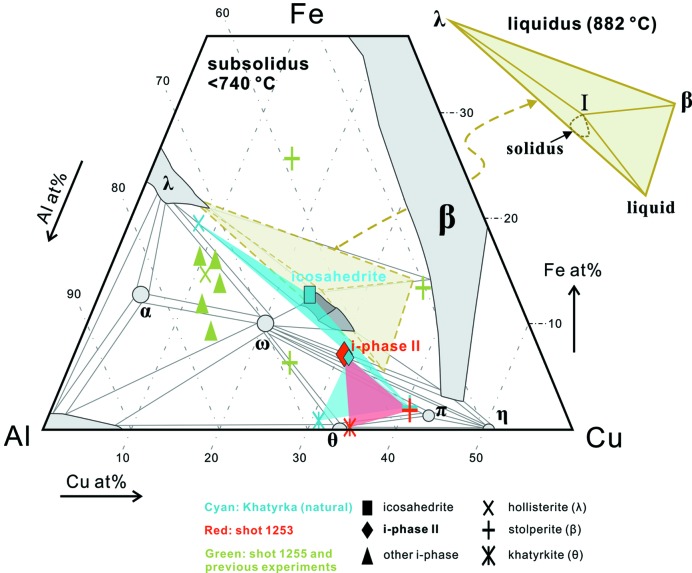
Representative subsolidus phase diagram of the tenary Al–Cu–Fe system at ambient pressure after the work by Bancel (1999 ▸). Light grey marks one-phase fields, and tie-lines delimit two-phase regions and three-phase triangles. Dark grey marks the field of icosahedrite. The light brown dash-line triangle indicates the area of the composition space shown in the upper-right inset for a section of the liquidus phase diagram at 882°C after the work by Zhang & Lück (2003 ▸). Against this background, data points are shown for the synthetic and natural phase assemblages discussed in this work; symbol shape represents the phase type and colour represents its origin. The contents of minor elements such as Ni and Cr are not reflected in this diagram. The coloured fields connect the co-existing phases in the icosahedrite and i-phase II assemblages. In the Khatyrka meteorite, icosahedrite co-exists with hollisterite and stolperite and defines the dark cyan obtuse triangle. In the same meteorite, i-phase II co-exists with khatyrkite and hollisterite and defines the light cyan triangle. The red field constrained by the shock-synthesized i-phase II assemblage in S1253 is close to that from Khatyrka. The i-phases from other shock experiments are concentrated on the more Cu-deficient side of the diagram.

Fig. 6 ▸ shows a representative isothermal section of the Al–Cu–Fe system at subsolidus temperature (<740 °C) and ambient pressure (Bancel, 1999 ▸). In this diagram, icosahedrite, i-phase II, hollisterite (λ), stolperite (β) and khatyrkite (θ) have been observed in the QC assemblage in the Khatyrka meteorite and in this study. A section of the liquidus diagram indicated by the brown triangle in Fig. 6 ▸ is also shown as an inset (Zhang & Lück, 2003 ▸). The liquidus triangle includes the distributory peritectic four-phase reaction point, stolperite + hollisterite + melt ⇌ icosahedrite, at 882°C. This is in good agreement with the occurrence of icosahedrite and directly associated hollisterite + stolperite in Khatyrka (Bindi *et al.*, 2016 ▸). Because the liquidus temperatures of hollisterite and stolperite are higher than that of icosahedrite (Bancel, 1999 ▸), icosahedrite rarely crystallizes directly from the melt. Instead, it forms by peritectic reaction between stolperite + hollisterite and evolving residual melt. In Khatyrka, both icosahedrite and i-phase II are enclosed within stolperite grains [Fig. 3 ▸(*d*); Bindi *et al.*, 2016 ▸]. Since i-phases are not supposed to form prior to stolperite, it is inferred that stolperite-associated icosahedrite and i-phase II both formed by peritectic reactions involving stolperite. For the i-phase II assemblage in S1253 [Fig. 3 ▸(*b*)], although i-phase II is not completely enclosed, it is partially surrounded by stolperite, commonly with concave boundaries. It is possible that the i-phase II in S1253 also formed by peritectic reaction with melt and stolperite. Now, unlike the pervasive stolperite, hollisterite is quite rare in the vicinity of both natural and shock-synthetic i-phase II [Figs. 3 ▸(*c*) and 3 ▸(*d*)]. In fact, khatyrkite is the other major constituent in the QC assemblages. This can best be explained by the fact that the i-phase II region has relatively low Fe content and hollisterite is almost completely consumed to provide the needed Fe for i-phase II (Table 1 ▸ and Fig. 6 ▸). That is, hollisterite appears to be the limiting reactant for the extent of formation of i-phase II. Subsequently, at lower temperature, <591°C, the residual melt reacts with stolperite to form khatyrkite through another peritectic reaction (Zhang & Lück, 2003 ▸; Suttle *et al.*, 2019 ▸). The khatyrkite predominant region in S1253 [Figs. 3 ▸ and S2(*c*)], only a few µm away from the i-phase II, is evidence that local Al–Cu melts with no Fe source can be supercooled to 591°C to make khatyrkite before any significant crystallization.

This two-stage peritectic reaction sequence is proposed to explain the texture and assemblage of i-phase II occurrences. It is still a question whether the observed assemblage can co-exist stably or if instead it is an evolving assemblage preserved metastably. In previous studies of subsolidus equilibrium at ambient pressure, the most ‘i-phase II-like’ compositions are Al_62.3_Cu_28.6_Fe_9.1_ annealed from 660°C (Zhang *et al.*, 2005 ▸) and Al_67–56_Cu_24–31_Fe_9–13_ annealed from 700°C (Zhu *et al.*, 2020 ▸). Nevertheless, these compositions are still not as Fe deficient as i-phase II. Moreover, the authors inferred a lower limit to their Fe content of i-phase co-existing with stolperite and khatyrkite assemblage from the topology of the phase diagram (Zhu *et al.*, 2020 ▸). In other words, if one draws a tie-line connecting i-phase II and hollisterite (instead of khatyrkite) on, for example, Fig. 6 ▸, the line would traverse the icosahedrite region, preventing these two phases from co-existing. However, the inferred khatyrkite-bearing assemblage was not actually observed in the vicinity of the Fe-deficient i-phases in previous experiments and the stability field of such an i-phase was not fully defined (Fig. 6 ▸). In this study and in Khatyrka (Bindi *et al.*, 2009 ▸, 2011 ▸, 2016 ▸), two assemblages are indeed observed that perfectly separate into two fields and do not interact with each other: (1) icosahedrite + hollisterite + stolperite and (2) i-phase II + stolperite + khatyrkite (Fig. 6 ▸). A simple explanation for their co-existence is that either or both the strong shear-stress field and >20 GPa pressure during a shock event cause the stability field of the i-phase to separate or shift towards Fe-deficient compositions and enables a peritectic crystallization sequence leading to the Fe-deficient i-phase assemblage, given a suitable starting composition. The compositional range for making i-phase II can be very narrow. The GDI was a successful starting material here because it traverses the full range of Al–Cu binary compositions. Even so, the extent of inflow of the steel chamber material needs to be coincidently right in order to avoid crossing into the other, more Fe-rich, i-phase field. It is also worth noting the possibility of i-phase II being a metastable phase at the relevant pressure–temperature conditions; its formation could still cause the separation of iron-rich and iron-deficient assemblages. In this scenario, the shock pressure may lower the activation energy for nucleation and growth of i-phase II and make it easier to form than under ambient-pressure conditions. Although there are not enough thermodynamic data on high-pressure Al–Cu–Fe i-phases to assess this hypothesis directly, experiments on a loosely analogous Zr_65_Al_7.5_Ni_10_Cu_7.5_Ag_10_ metallic glass indicate that the energy barrier for QC formation decreases by 40% at 0.86 GPa compared with ambient pressure (Jiang *et al.*, 2001 ▸). Subsequent fast quenching during a shock event would then be advantageous for preserving the metastable assemblage.

In contrast to S1253, the i-phase + hollisterite + stolperite assemblage in S1255 shows a texture of simple crystallization, more like an equilibrium rather than peritectic assemblage. The Al_68.6_Cu_11.2_Fe_14.5_Cr_4_Ni_1.8_ composition of the i-phase matches with the Al_68–73_Fe_11–16_Cu_10–12_Cr_1–4_Ni_1–2_ composition seen in previous experiments on CuAl_5_ alloys. The current data are consistent with a separation of the stability field of i-phase into two fields: Fe deficient as in S1253 and Fe rich as in S1255. However, considering that the composition of icosahedrite does not change much with the application of static high pressure (Stagno *et al.*, 2014 ▸, 2015 ▸), a possible alternative is that i-phase I is stabilized by the presence of Cr + Ni whereas i-phase II forms and quenches metastably through the proposed two-stage peritectic reaction sequence.

### 4.3. Impact conditions recorded by the Khatyrka meteorite   

The near-exact reproduction of the natural i-phase II assemblage in S1253 helps to better constrain the impact conditions experienced and recorded by the Khatyrka meteorite. The finding of ahrensite (from transformation) and stishovite (crystallized from melt) in the silicate lithology of the Khatyrka meteorite has supported inference of a shock-pressure well above 5 GPa, likely to be ∼20 GPa for one shock event (Hollister *et al.*, 2014 ▸; Ma *et al.*, 2016 ▸). The shock pressure for the reaction zone of interest in S1253 is 20–25 GPa (Fig. S1). The 5 GPa difference between these results is not a discrepancy because the experiment has not placed a lower bound on the shock pressure for formation of the i-phase II assemblage. Nevertheless, if high shock pressure and shear stress truly shifts or separates the stability field of the i-phase and determines whether the Fe-rich or the Fe-deficient assemblage forms, then icosahedrite would tend not to form at the same conditions as i-phase II. In a single shock scenario, i-phase II and its assemblage would occur from material quenched during the high-pressure pulse, whereas material that remains as hot Al–Cu–Fe melt until after the shock-pressure release could crystallize icosahedrite and its co-existing assemblage. In contrast, the formation of the Al–Cu metal precursors to all the shock-induced reaction chemistry described here probably requires a separate earlier event, as shown by the petrographic evidence reported in the work of Lin *et al.* (2017 ▸) and by uranium Th–He dating of olivine in Khatyrka (Meier *et al.*, 2018 ▸).

## 5. Conclusions   

Al–Cu–W disks with graded composition (GDIs) are useful for sampling a wide range of Al/Cu ratios and efficiently exploring composition space for conditions that may yield variant Al–Cu–Fe quasicrystals in shock-recovery experiments. With our experiments, the naturally occurring i-phase II quasicrystal (Al_62_Cu_31_Fe_7_) found in the Khatyrka meteorite has been reproduced for the first time in a laboratory setting, along with the associated natural assemblage of stolperite plus khatyrkite. Another more Fe-rich quinary i-phase (Al_68.6_Fe_14.5_Cu_11.2_Cr_4_Ni_1.8_) is also produced together with stolperite and hollisterite. The shock synthesis of the i-phase II + stolperite + khatyrkite assemblage suggests that the thermodynamic stability field of icosahedrite (Al_63_Cu_24_Fe_13_) could shift and/or separate under shock conditions above 20 GPa, leading to the co-existence of Fe-rich and Fe-poor i-phases like the case in Khatyrka. Future experiments, for example with Cr- and Ni-free starting material, will better resolve the phase boundaries of Al–Cu–Fe i-phases under impact conditions.

## Supplementary Material

Supporting information. DOI: 10.1107/S2052252520002729/lt5026sup1.pdf

## Figures and Tables

**Table 1:** Compositions of the intermetallic phases in S1253 and the Khatyrka meteorite Data from this study (S1253) and the Khatyrka meteorite are presented in atomic percent and normalized to a total of 100%. Numbers of analyses are in parentheses. Absent numbers are for elements below the detection limit or not reported. Variance of the analyses is presented using standard deviation of the mean (σ). The data for i-phase II are averaged from analyses under 10 kV accelerating voltage using the SEM. Results for individual analyses are shown in Table S1.

	i-phase II		Khatyrkite		Stolperite	
Element	S1253 (9)	Khatyrka† ‡	S1253 (3)	Khatyrka§	S1253 (7)	Khatyrka§
Al	61.47	61.92	65.09	68.48	57.35	57.34
Cu	30.30	31.23	34.63	30.83	40.56	40.58
Fe	6.80	6.78	0.28	0.69	1.57	2.08
Cr	1.42	0.08	—	—	0.40	—
Ni	—	—	—	—	0.12	—
σ						
Al	1.33	0.99	1.00	1.78	1.26	1.33
Cu	2.82	0.80	1.03	0.80	1.21	0.94
Fe	1.69	0.37	0.24	0.02	0.61	0.05
Cr	0.34	0.02	—	—	0.21	—
Ni	—	—	—	—	0.20	—

† Bindi *et al.* (2016 ▸).

‡ Lin *et al.* (2017 ▸).

§ Ma *et al.* (2017 ▸).

## References

[bb1] Asimow, P. D., Lin, C., Bindi, L., Ma, C., Tschauner, O., Hollister, L. S. & Steinhardt, P. J. (2016). *Proc. Natl Acad. Sci. USA*, **113**, 7077–7081. 10.1073/pnas.1600321113 PMC4932946 27298357

[bb2] Bancel, P. A. (1999). *Quasicrystals: The State of the Art*, 2nd ed., edited by D. P. DiVincenzo & P. J. Steinhardt, Vol. 16, pp. 17–55. Singapore: World Scientific.

[bb3] Bindi, L., Eiler, J. M., Guan, Y., Hollister, L. S., MacPherson, G., Steinhardt, P. J. & Yao, N. (2012). *Proc. Natl Acad. Sci. USA*, **109**, 1396–1401. 10.1073/pnas.1111115109 PMC3277151 22215583

[bb4] Bindi, L., Lin, C., Ma, C. & Steinhardt, P. J. (2016). *Sci. Rep.* **6**, 38117. 10.1038/srep38117 PMC5144682 27929519

[bb5] Bindi, L., Steinhardt, P. J., Yao, N. & Lu, P. J. (2009). *Science*, **324**, 1306–1309. 10.1126/science.1170827 19498165

[bb6] Bindi, L., Steinhardt, P. J., Yao, N. & Lu, P. J. (2011). *Am. Mineral.* **96**, 928–931.

[bb7] Bindi, L., Yao, N., Lin, C., Hollister, L. S., Andronicos, C. L., Distler, V. V., Eddy, M. P., Kostin, A., Kryachko, V., MacPherson, G. J., Steinhardt, W. M., Yudovskaya, M. & Steinhardt, P. J. (2015*a*). *Am. Mineral.* **100**, 2340–2343.

[bb8] Bindi, L., Yao, N., Lin, C., Hollister, L. S., Andronicos, C. L., Distler, V. V., Eddy, M. P., Kostin, A., Kryachko, V., MacPherson, G. J., Steinhardt, W. M., Yudovskaya, M. & Steinhardt, P. J. (2015*b*). *Sci. Rep.* **5**, 9111. 10.1038/srep09111 PMC4357871 25765857

[bb9] Gauvin, R., Lifshin, E., Demers, H., Horny, P. & Campbell, H. (2006). *Microsc. Microanal.* **12**, 49–64. 10.1017/S1431927606060089 17481341

[bb10] Hollister, L. S., Bindi, L., Yao, N., Poirier, G. R., Andronicos, C. L., MacPherson, G. J., Lin, C., Distler, V. V., Eddy, M. P., Kostin, A., Kryachko, V., Steinhardt, W. M., Yudovskaya, M., Eiler, J. M., Guan, Y., Clarke, J. J. & Steinhardt, P. J. (2014). *Nat. Commun.* **5**, 4040. 10.1038/ncomms5040 24925481

[bb11] Jiang, J. Z., Zhuang, Y. X., Rasmussen, H., Saida, J. & Inoue, A. (2001). *Phys. Rev. B*, **64**, 094208.

[bb12] Kelly, J. P., Nguyen, J. H., Lind, J., Akin, M. C., Fix, B. J., Saw, C. K., White, E. R., Greene, W. O., Asimow, P. D. & Haslam, J. J. (2019). *J. Appl. Phys.* **125**, 145902.

[bb13] Kipp, M. E. & Lawrence, R. J. (1982). NASA STI/Recon Technical Report N83.

[bb14] Lemmerz, U., Grushko, B., Freiburg, C. & Jansen, M. (1994). *Philos. Mag. Lett.* **69**, 141–146.

[bb15] Lifshitz, R. (2003). *Found. Phys.* **33**, 1703–1711.

[bb16] Lin, C., Hollister, L. S., MacPherson, G. J., Bindi, L., Ma, C., Andronicos, C. L. & Steinhardt, P. J. (2017). *Sci. Rep.* **7**, 1637. 10.1038/s41598-017-01445-5 PMC5431614 28487537

[bb17] Ma, C., Lin, C., Bindi, L. & Steinhardt, P. J. (2017). *Am. Mineral.* **102**, 690–693.

[bb18] Ma, C., Tschauner, O., Beckett, J. R., Liu, Y., Rossman, G. R., Sinogeikin, S. V., Smith, J. S. & Taylor, L. A. (2016). *Geochim. Cosmochim. Acta*, **184**, 240–256.

[bb19] MacPherson, G. J., Andronicos, C. L., Bindi, L., Distler, V. V., Eddy, M. P., Eiler, J. M., Guan, Y., Hollister, L. S., Kostin, A., Kryachko, V., Steinhardt, W. M., Yudovskaya, M. & Steinhardt, P. J. (2013). *Meteorit. Planet. Sci.* **48**, 1499–1514.

[bb20] Meier, M. M. M., Bindi, L., Heck, P. R., Neander, A. I., Spring, N. H., Riebe, M. E. I., Maden, C., Baur, H., Steinhardt, P. J., Wieler, R. & Busemann, H. (2018). *Earth Planet. Sci. Lett.* **490**, 122–131.

[bb21] Oppenheim, J., Ma, C., Hu, J., Bindi, L., Steinhardt, P. J. & Asimow, P. D. (2017*a*). *Sci. Rep.* **7**, 15628. 10.1038/s41598-017-15229-4 PMC5688080 29142198

[bb22] Oppenheim, J., Ma, C., Hu, J., Bindi, L., Steinhardt, P. J. & Asimow, P. D. (2017*b*). *Sci. Rep.* **7**, 15629. 10.1038/s41598-017-15229-4 PMC5688080 29142198

[bb23] Porter, D. A., Easterling, K. E. & Sherif, M. (2009). *Phase Transformations in Metals and Alloys*. 3rd ed. Boca Raton: CRC Press.

[bb24] Potter, D. K. & Ahrens, T. J. (1994). *Geophys. Res. Lett.* **21**, 721–724.

[bb25] Shechtman, D., Blech, I., Gratias, D. & Cahn, J. W. (1984). *Phys. Rev. Lett.* **53**, 1951–1953.

[bb26] Stagno, V., Bindi, L., Park, C., Tkachev, S., Prakapenka, V. B., Mao, H.-K., Hemley, R. J., Steinhardt, P. J. & Fei, Y. (2015). *Am. Mineral.* **100**, 2412–2418.

[bb27] Stagno, V., Bindi, L., Shibazaki, Y., Tange, Y., Higo, Y., Mao, H.-K., Steinhardt, P. J. & Fei, Y. (2014). *Sci. Rep.* **4**, 5869. 10.1038/srep05869 PMC5376180 25070248

[bb28] Stagno, V., Bindi, L., Steinhardt, P. J. & Fei, Y. (2017). *Phys. Earth Planet. Inter.* **271**, 47–56.

[bb29] Steurer, W. & Deloudi, S. (2009). *Crystallography of Quasicrystals*. Berlin, Heidelberg: Springer-Verlag.

[bb30] Suttle, M. D., Twegar, K., Nava, J., Spiess, R., Spratt, J., Campanale, F. & Folco, L. (2019). *Sci. Rep.* **9**, 1–9. 10.1038/s41598-019-48937-0 PMC6711995 31455844

[bb31] Tsai, A.-P. (1999). *Physical Properties of Quasicrystals*, edited by Z. M. Stadnik, Vol. 126, pp. 5–50. Berlin, Heidelberg: Springer-Verlag.

[bb32] Tsai, A.-P., Inoue, A. & Masumoto, T. (1987). *Jpn J. Appl. Phys.* **26**, L1505–L1507.

[bb33] Wolf, W., Kube, S. A., Sohn, S., Xie, Y., Cha, J. J., Scanley, B. E., Kiminami, C. S., Bolfarini, C., Botta, W. J. & Schroers, J. (2019). *Sci. Rep.* **9**, 7136. 10.1038/s41598-019-43666-w PMC6509252 31073200

[bb35] Zhang, L. & Lück, R. (2002). *J. Alloys Compd.* **342**, 53–56.

[bb36] Zhang, L. & Lück, R. (2003). *Z. Metallkd.* **94**, 91–97.

[bb34] Zhang, L., Schneider, J. & Lück, R. (2005). *Intermetallics*, **13**, 1195–1206.

[bb37] Zhu, L., Soto-Medina, S., Cuadrado-Castillo, W., Hennig, R. G. & Manuel, M. V. (2020). *Mater. Des.* **185**, 108186.

