# Supplementary material for: First synthesis of a unique icosahedral phase from the Khatyrka meteorite by shock-recovery experiment
Source: IUCrJ. 2020 Mar 26;7(Pt 3):434–44. doi: 10.1107/S2052252520002729 (PMC7201281; doi:10.1107/S2052252520002729)
Supplement: Supplementary file 1 [file m-07-00434-sup1.pdf]

IUCrJ

Volume 7 (2020)

Supporting information for article:

First synthesis of a unique icosahedral phase from the Khatyrka meteorite by shock-recovery experiment

Jinping Hu, Paul D. Asimow, Chi Ma and Luca Bindi

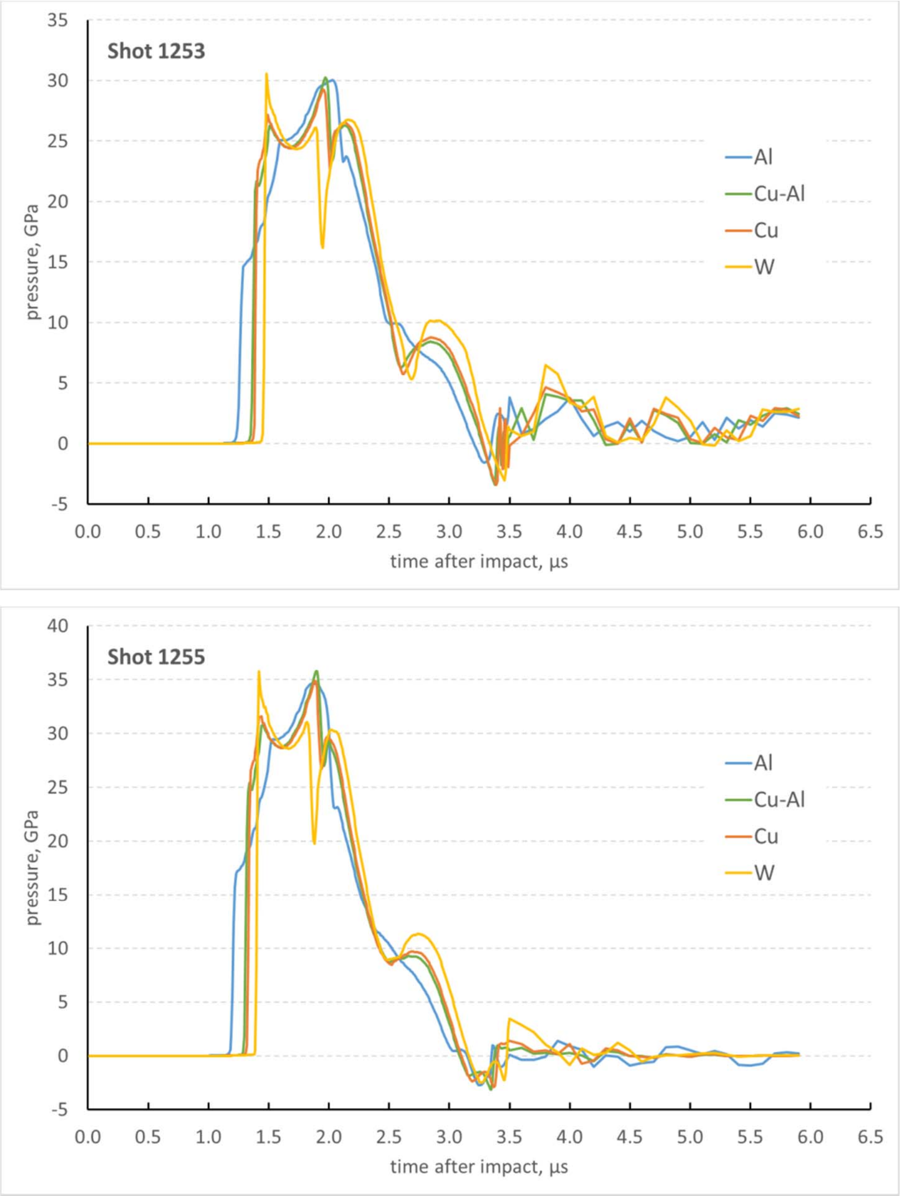

Figure S1 Shock pressure history in the layers of the Al-Cu-W GDI sample for shot 1253 (Ta flyer, 0.93 km/s impact velocity) and 1255 (SS304 flyer, 1.28 km/s). Calculated by Wondy 1-D hydrocode. The calculation is based on the full layers of Al-Cu-W stack in the thick part of the GDI wedge and used material properties in Kelly *et al*. (2019). See Section 2.2 in the main text for details.

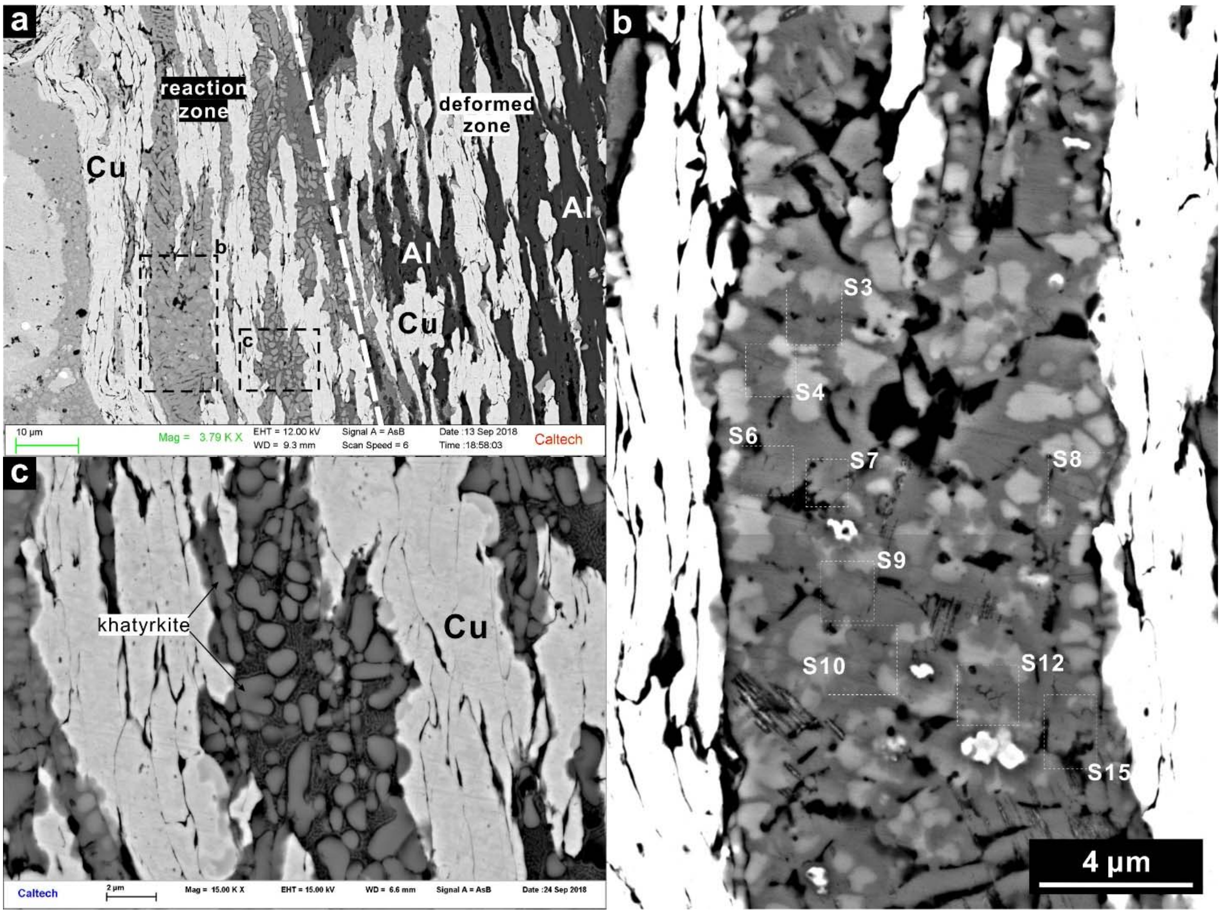

Figure S2 The occurrences of Al-Cu-Fe quasicrystals with intermetallic phases in S1253. (a) A SEM BSE image showing the strongly deformed Al and Cu grains adjacent to the melted reaction zone. In the reaction zone, all the Al grains are consumed and remnant Cu grains co-exist with intermetallic phases. Boxed areas are enlarged in b and c. (b) The i-phase II + khatyrkite + stolperite assemblage. High magnification images of these phases are in Figure 3 and S3. White boxes mark the most well-defined i-phase II domains. The chemical composition of each grain is in Table S1. (c) A khatyrkite assemblage that excludes i-phase. The interstitial phases are too fine to be determined, inferred to be mixture of Al-rich alloys. The composition of the khatyrkite is in Table S2.

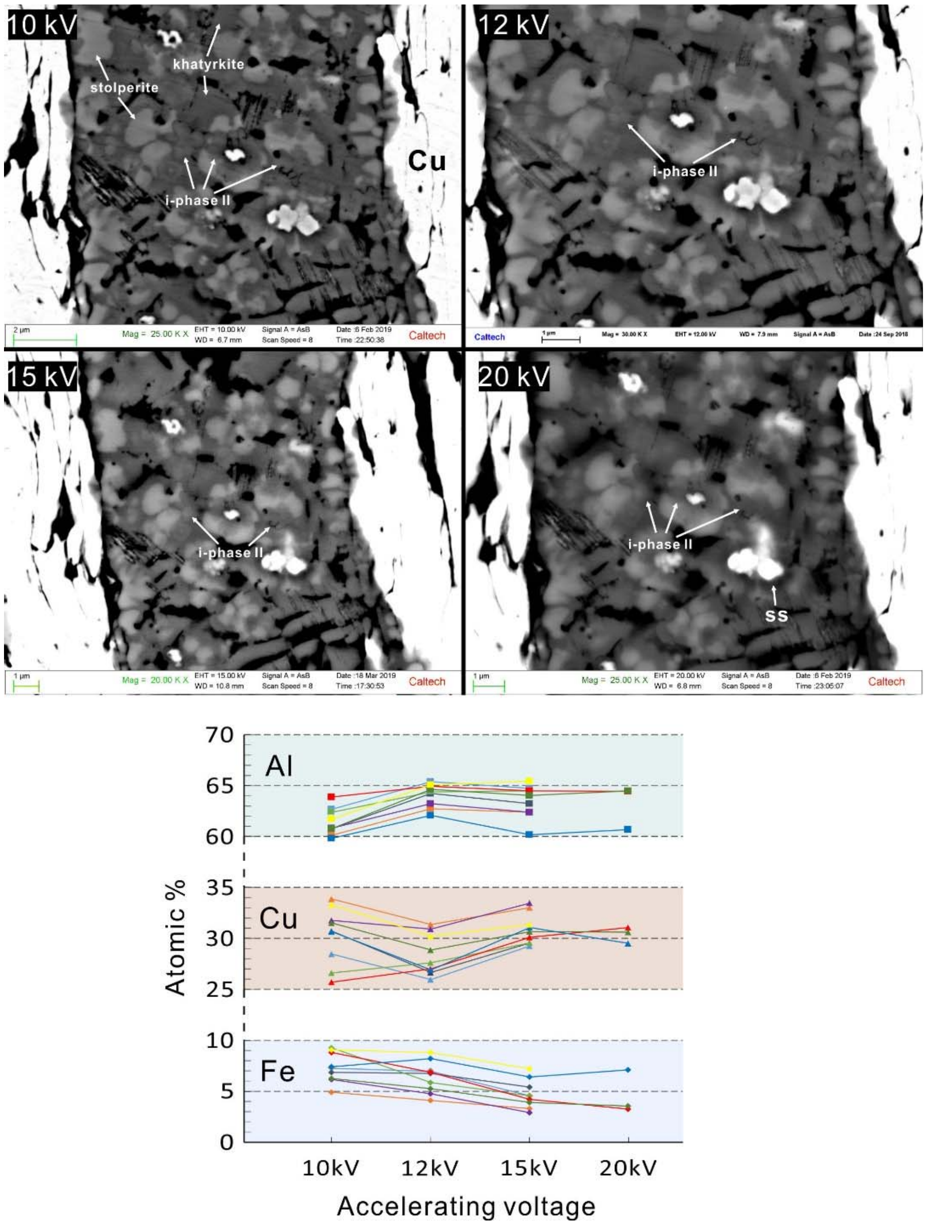

Figure S3 BSE images of the i-phase II bearing area using different SEM accelerating voltages. The high-voltage setup has larger excitation volume and the corresponding image shows more subsurface information. At 20 kV, the steel (ss) fragment at subsurface stands out more whereas the grain boundaries of submicron quasicrystal domains become almost indistinguishable. The bottom figure is the compositions from EDS analysis of individual i-phase II domains using different accelerating voltages. Line connects the results from the same domain. Corresponding data are in Table S1.

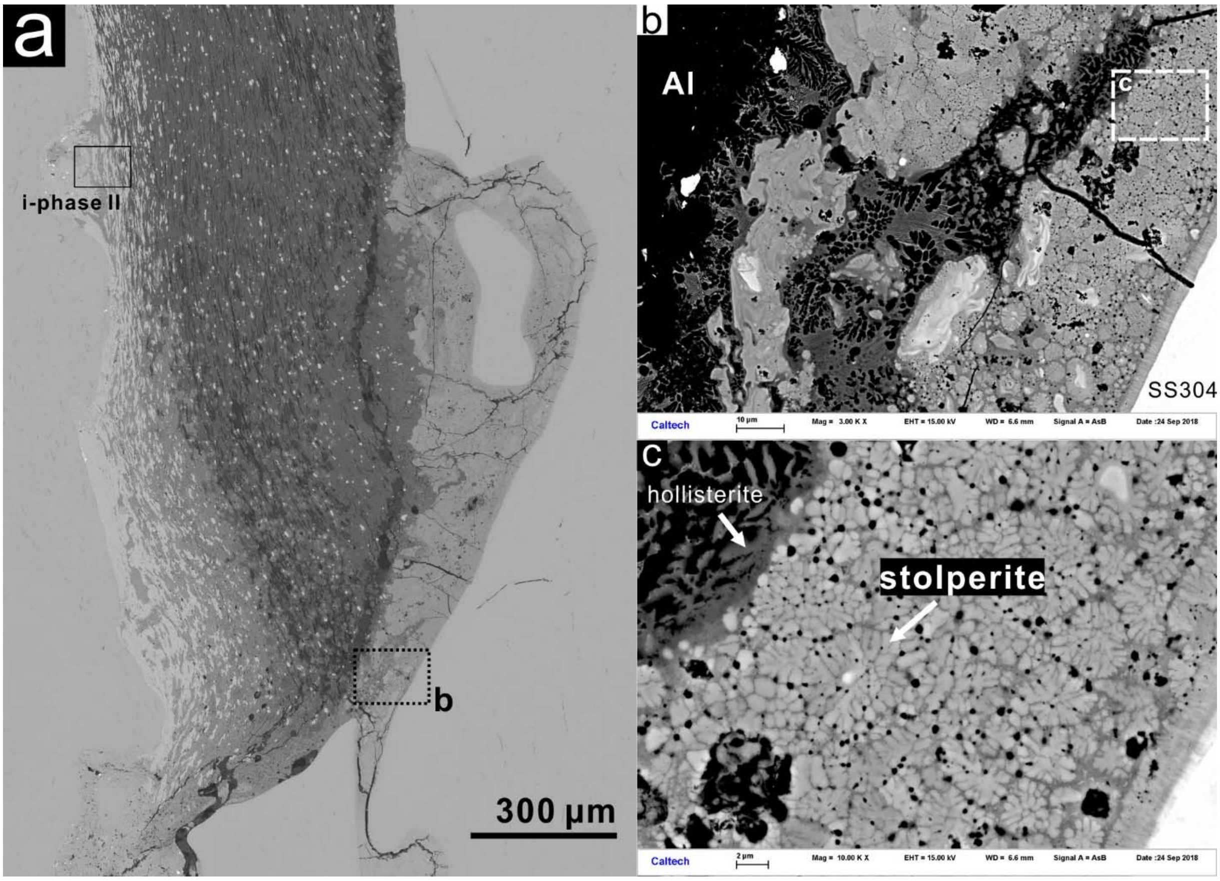

Figure S4 BSE images of the reaction zones on the Al-rich side of the GDI. (a) Overall image for the strong deformed corner of the GDI wedge. The i-phase II region on the Cu-rich side is presented in Figure 3, S2 and S3. The Al-rich side is enlarged in b. (b) The assemblage with distinctively darker Al-rich phases and brighter Fe-rich phases adjacent to the steel. The remnant Al grains are almost black at this contrast. (c) The radial pattern of stolperite aggregates. Mixture of remnant Al and hollisterite is on the upper-left corner. The compositions of the phases are shown in Table S2.

Table S1 EDS analysis of i-phase II domains in shot 1253

Domain	Accelerating Voltage	Al	Cu	Fe	Cr	Ni
S3	10 kV	62.71	28.47	7.30	1.53	
	12 kV	65.43	25.98	6.99	1.61	
	15 kV	64.74	29.30	4.57	1.06	0.34
S4	10 kV	60.16	33.87	4.91	1.06	
	12 kV	62.75	31.39	4.13	1.15	0.58
	15 kV	62.39	33.05	3.35	0.89	0.31
S6	10 kV	60.81	30.73	6.90	1.56	
	12 kV	64.27	26.67	6.79	1.50	0.77
	15 kV	63.27	29.56	5.45	1.18	0.54
S7	10 kV	62.42	26.60	9.29	1.69	
	12 kV	64.41	27.64	5.86	1.41	0.68
	15 kV	64.48	29.58	4.56	1.08	0.30
S8	10 kV	60.84	31.76	6.16	1.24	
	12 kV	63.25	30.91	4.77	1.06	
	15 kV	62.41	33.47	2.95	0.73	0.43
S9	10 kV	63.92	25.74	8.86	1.48	
	12 kV	64.93	27.04	6.86	1.16	
	15 kV	64.51	30.15	4.25	0.84	0.25
	20 kV	64.47	31.08	3.29	0.78	0.37
S10	10 kV	60.81	31.55	6.29	1.35	
	12 kV	64.64	28.87	5.30	1.19	
	15 kV	64.03	30.69	3.91	0.98	0.40
	20 kV	64.49	30.62	3.58	0.97	0.35
S12	10 kV	59.86	30.70	7.42	2.02	
	12 kV	62.10	26.90	8.23	1.88	0.89
	15 kV	60.21	31.06	6.44	1.76	0.54
	20 kV	60.71	29.52	7.15	1.93	0.69
S15	10 kV	61.74	33.32	4.07	0.87	
	12 kV	65.11	30.21	3.83	0.85	
	15 kV	65.48	31.40	2.22	0.65	0.25
Averages		Al	Cu	Fe	Cr	Ni
	10kV	61.47	30.30	6.80	1.42	-
	σ	1.33	2.82	1.69	0.34	-
	12kV	64.10	28.40	5.86	1.31	0.32
	σ	1.14	2.01	1.47	0.32	0.39
	15kV	63.50	30.92	4.19	1.02	0.37
	σ	1.62	1.51	1.28	0.33	0.11

Data are presented in atomic percent and normalized to 100%. The uncertainties for the averages are calculated in standard deviations. Each spot is analyzed on SEM-EDS with 10, 12 and 15 kV accelerating voltages, of which three are analyzed at 20 kV. The analyzed domains are shown in Figure S2.

Table S2 Composition of Al-Cu-Fe intermetallic phases in non-QC regions in Shot 1253

Occurrence	Phase	Al	Cu	Fe	Cr	Ni	Mn	Si
Fig. S2c	Khatyrkite	71.28	28.72					
		67.25	32.75					
Fig. S4c	Hollisterite	64.67	8.15	18.71	5.48	2.51	0.47	
	Stolperite	48.96	7.6	30.31	9.04	3.24	0.54	0.31

Data are presented in atomic percent and normalized to 100%. Corresponding textures of the phases are shown in Figure S2c and S4c.

Table S3 Compositions of i-phase and intermetallic phases in Shot S1255

Phase	Al	Cu	Fe	Cr	Ni
i-phase	70.07	10.57	14.10	3.73	1.52
	66.56	11.48	15.92	4.17	1.87
	67.19	9.27	17.15	4.66	1.73
	68.21	11.63	14.49	4.18	1.50
	70.09	11.74	13.06	3.42	1.68
	67.71	11.68	13.80	4.45	2.36
	66.96	11.14	15.62	4.20	2.08
	71.68	12.12	11.53	3.16	1.52
average	68.56	11.20	14.46	4.00	1.78
σ	1.84	0.91	1.77	0.51	0.31
Stolperite	47.54	34.47	12.69	2.92	2.38
	53.44	14.13	22.97	6.69	2.77
	58.45	24.71	12.12	3.41	1.32
Hollisterite	70.43	10.58	13.68	3.89	1.42

The compositions are shown in atomic percent and normalized to 100%. The uncertainty is presented in standard deviation (σ). Occurrences of the phases are shown in Figure 4.

Table S4 Compositions of icosahedral phases in shock experiments and the Khatyrka meteorite

	S1253 i-phase II (this study)	natural i-phase II[1,2]	natural icosahedrite (i-phase I)[1,3]	S1255 i-phase (this study)	Previous shock synthetic[4]	Previous shock synthetic[4]	Previous shock synthetic[4]	Previous shock synthetic[5]
Al	61.47	61.92	63.19	68.56	72.30	73.30	67.94	70.3
Cu	30.30	31.22	24.23	11.20	9.70	11.57	11.30	11.5
Fe	6.80	6.78	12.55	14.46	15.70	11.30	15.42	13.2
Cr	1.42	0.08	0.03	4.00	1.02	2.73	3.73	3.7
Ni	-	-	-	1.78	1.23	1.11	1.62	1.3
Cr/Fe	0.21	0.01	-	-	0.06	0.24	0.24	0.28
Ni/Cr	-	-	-	-	1.21	0.41	0.43	0.40
Uncertainties								
Al	1.33	0.99	0.33	1.84	0.50	0.40	0.13	2.30
Cu	2.82	0.80	0.55	0.91	0.10	0.05	0.06	1.00
Fe	1.69	0.37	0.71	1.77	0.50	0.30	0.13	1.00
Cr	0.34	0.02	0.03	0.51	0.03	0.06	0.05	0.40
Ni				0.31	0.03	0.05	0.05	0.20

Data are presented in atomic percentage and normalized to 100%. Uncertainties are presented in standard deviation. Absent numbers are for elements below detection limit or not reported. [1]Bindi et al. 2016, [2]Lin et al. 2017, [3]Bindi et al. 2011, [4]Asimow et al. 2016, [5]Oppenheim et al. 2017a
